# Does personal relevance moderate communication effects? The example of risk communication about 5G-related electromagnetic fields.

**DOI:** 10.12688/openreseurope.19236.1

**Published:** 2025-01-20

**Authors:** Marie Eggeling-Böcker, Efthymios Karabetsos, Maria Christopoulou, Sarah C. Link, Ferdinand Abacioglu, Christoph Boehmert

**Affiliations:** 1 IU International University of Applied Sciences, Erfurt, Germany; 2 Greek Atomic Energy Commission, Athens, Attica, Greece

**Keywords:** personal relevance, risk communication, precaution, mobile communications, RF-EMF, 5G, risk perception

## Abstract

**Background:**

Technological advancements, such as the introduction of 5G, offer opportunities but also raise concerns. Although no evidence suggests negative effects of radiofrequency electromagnetic fields (RF-EMF) within defined exposure limits, authorities responsible for risk communication provide precautionary advice to help citizens reduce personal exposure. However, previous research indicates that precautionary information can increase risk perception and decrease trust.

**Methods:**

This cross-sectional study investigated effects of precautionary information on risk perception and trust in the context of 5G, using large general population samples in Germany and Greece. For the first time, personal relevance was examined as a potential moderating factor, using a novel approach to assess practical and thematic relevance. Participants were first surveyed on their relevance of the topic, then provided with basic information about “RF-EMF and health”, and, in the experimental group, with additional precautionary information. Different measures for risk perception and trust followed. We expected higher risk perception and lower trust for the experimental group, and assumed that at lower personal relevance, this effect would be stronger.

**Results:**

Contrary to expectations, precautionary information increased just one risk perception measure and only in Germany. The anticipated moderating effect of personal relevance was not found, but relevance itself significantly predicted risk perception, with higher relevance correlating with higher risk perception. Exploratory findings revealed higher risk perception among females compared to males and in Greece compared to Germany.

**Conclusions:**

That there were only few effects of the precautionary information may be linked to the focus on actions to reduce personal exposure when using mobile devices. The results underline the importance of considering personal relevance and demographic factors in risk communication and highlight directions for future research.

## Plain Language Summary

Advances in technology, like 5G in mobile communications, bring both opportunities and concerns. There is no evidence that mobile phone radiation (electromagnetic fields) harms health when kept within safety limits. Nevertheless, authorities often give information on how they can reduce their exposure if they wish to do so (precautionary information). However, such information can sometimes make people more worried and less trusting.

In this study we tested whether providing precautionary information about mobile phone radiation and 5G affects how risky people think it is and how much they trust in authorities providing it. Participants came from Germany and Greece, and they took part in the study online. First, they were asked how much the topic “mobile phone radiation and health” mattered to them. Then, one group of participants received only general information on the topic and the other one received extra precautionary information. We then asked them for their perceptions of risk (for example, how worried they feel about the topic) and trust. We expected that people who read the precautionary information would perceive higher risk and experience less trust than people who read the basic information. Also, we expected that people who cared less about the topic might be more affected by the precautionary information.

Unexpectedly, we found that for the most part the group that read precautionary information did not perceive them as more risky or less trustworthy than the group that read basic information. People to whom the topic mattered more perceived it as riskier. However, there was no difference in how they perceived the precautionary information. The study also found that Greek participants compared to German ones and women compared to men perceived the topic as riskier.

In summary, precautionary information had little effect on risk perception or trust. Personal relevance and other individual differences, like gender and nationality, played a role in how people viewed the risks. This is important to consider for people who inform the public about risks and health-related topics.

## Glossary

RF-EMF Radiofrequency Electromagnetic Fields

GHz       Gigahertz

kHz        Kilohertz

WHO     World Health Organization

IARC      International Agency for Research on Cancer

ICNIRP   International Commission for Non-Ionizing Radiation Protection

IEEE       Institution of Electrical and Electronic Engineers

BfS         Bundesamt fuer Strahlenschutz (the German national radiation protection agency)

EEAE     Greek Atomic Energy Commission (the Greek national radiation protection agency)

RP          Risk Perception

MC         Mobile Communication

MP         Mobile Phone

BS         Base Station

CR1      (General) Conditional risk perception assuming that no precautions are taken

CR2      (General) Conditional risk perception assuming that precautions are taken

## Introduction

Technological advancements, for example in the field of mobile communication (MC) technology, bring along many possibilities and benefits, but also concerns about risks, often involving potential health, environmental, and societal impacts that are not immediately apparent. As technologies are advancing fast, such consequences may emerge over time, making it challenging to predict and manage their full effects.

In response to these challenges, the precautionary principle has become an important approach, particularly when scientific uncertainty limits our understanding of potential risks, for example because there is not enough research regarding the topic or because scientific evidence is inconclusive ( Klinke & Renn, 2001). Precautionary measures can be implemented by governments or health authorities, and they can also be shared with the public, giving individuals the option to adopt them voluntarily ( Wiedemann *et al.*, 2001). For risk communicators it is important to understand how information about precautionary measures is perceived by the citizens.

One area where precautions, and particularly the effects of communicating about them to the public, has been a topic of discussion, is the field of MC, and particularly the new fifth generation technology standard 5G. Recommended precautionary actions in this field concern reducing personal exposure to radiofrequency electromagnetic fields (RF-EMF). However, experimental studies have found that providing precautionary information can result in higher risk perception (RP) and lower trust compared to providing basic information only. In the study presented in this paper, the effects of precautionary information are investigated with a large general population sample, considering the introduction of 5G, and for the first time including personal relevance of the topic as a possible influencing factor.

### The case of 5G, RF-EMF and health

Exposure to RF-EMF at frequencies from 100 kHz to 300 GHz has been steadily increasing during the last 70 years, with primarily radio and television signals and, more recently, including wireless telecommunications and other applications (e.g., industrial and medical). Due to the ubiquitous and increasing exposure to RF-EMF, the question, if RF-EMF from MC-technology have negative impacts on human health, has been researched for many years now. So far, the only biological effects that have been consistently found are the thermal effects, meaning the heating of tissues for whole body or for partial body (local) exposures ( Foster & Colombi, 2017; ICNIRP, 2020; IEEE, 2019). Other effects that may be negative for human health have not been consistently found ( Wood & Karipidis, 2017). This is the case for both older MC-standards as well as for the latest standard 5G ( SCHEER, 2023; Udo *et al.*, 2022). The World Health Organization (WHO) has an ongoing project to update the assessment regarding potential health effects of exposure to RF-EMF in the general and working population. The analysis and synthesis of available evidence will be published as a monograph in the WHO Environmental Health Criteria (EHC) series. To this end, WHO has commissioned a set of systematic reviews related to several health outcomes (cancer, adverse reproductive outcomes, cognitive impairment, and symptoms) and biological outcomes (heating and oxidative stress). Many of these systematic reviews are already published ( Ijaz *et al.*, 2024). The International Commission for Non-Ionizing Radiation Protection (ICNIRP) and the Institution of Electrical and Electronic Engineers (IEEE) recommend exposure limits for health protection, which many countries have adopted ( Missling *et al.*, 2015; Stam, 2017). These recommended limits are reviewed periodically in order to keep up with the latest scientific developments and research outcomes. Nevertheless, scientific uncertainties remain, e.g., regarding long-term effects on heavy users of MC-technology or effects on children ( World Health Organization, 2010; World Health Organization, 2014). In 2013, the International Agency for Research on Cancer (IARC) classified the RF-EMF emitted by mobile phones as “possibly carcinogenic to humans” (Group 2B), a category used when a causal association is considered credible, but when chance, bias or confounding cannot be ruled out with reasonable confidence ( IARC, 2013). This means that the risk of developing brain tumours for heavy mobile phone users cannot be ruled out by the current state of scientific evidence but may be also due to other factors.

### Communication of precautionary information to the public

According to the World Health Organization ( World Health Organization, 2012), their scope of responsibility contains only recommendations regarding prevention and does not include precaution, because there is no evidence for negative health effects from RF-EMF within the limits. They also argue that the recommendation of measures should depend on other factors (e.g., socio-economic reality) and be therefore left to national authorities. Many national health authorities (e.g., the Federal Office for Radiation Protection, BfS, in Germany ( BfS, 2024), the Greek Atomic Energy Commission, EEAE, in Greece ( EEAE, 2020), and the United States Environmental Protection Agency, EPA ( EPA, 2024) presented precautionary information on their websites at the time this study took place, often focusing on measures regarding mobile devices that citizens can decide to implement in their daily lives.

### Previous research on effects of precautionary information on risk perception and trust

A closer look at precautionary information reveals that it might be considered as inconsistent by those who receive the information. Why are people informed about actions to reduce exposure if there are no known health effects? The question how presenting precautionary information regarding RF-EMF in MC influences RP, i.e. the subjective evaluation of a (potential) hazard, influenced by the perceived probability of a hazard occurring and its expected severity ( Sjöberg *et al.*, 2004; Wilson *et al.*, 2019), and trust (i.e. in public health protection or the communicating institution) has been researched in several studies.

In 2020, Boehmert, Freudenstein, and Wiedemann conducted a systematic review to summarize the previous research on risk communication regarding RF-EMF in MC-technology. For precautionary recommendations, they found that across all studies, there was no significant effect on general RF-EMF RP, but recommendations led to significantly higher RP concerning specifically mobile phones and mobile phone base stations (small effect sizes). The effects regarding trust however were not consistent and they were measured less often. It is important to note that those studies differed regarding several aspects in their design and contents. For example, they either communicated precautionary measures that are implemented by authorities (like implementing stricter limit values for RF-EMF exposure, e.g., Wiedemann *et al.*, 2006; Wiedemann & Schütz, 2005; Wiedemann *et al.*, 2013) or measures one can take individually to reduce personal exposure when using a mobile phone (e.g., Boehmert *et al.*, 2016; Boehmert, 2018). In addition, there are indications that the effect of precautionary information is moderated by some variables, i.e., gender, country of origin, or general concern and anxiety. For example, Boehmert and colleagues ( Boehmert *et al.*, 2017; Boehmert, 2018) included gender and trait anxiety in their analyses. Though their results were not consistent, their findings indicate that females have a higher RP compared to males and that people with high trait anxiety are less affected by the precautionary information than people with low trait anxiety.

### Personal relevance of the topic

While the influence of some individual characteristics on the effects of precautionary information has been investigated, it has not yet been researched how the effect is related to personal relevance of the topic. To consider relevance would be particularly important if it moderated how different information affects RP and trust.

While relevance has to our knowledge not yet been examined as an influencing factor in studies on precautionary information in MC, a study on RP and exposure perception regarding EMF in general by Wiedemann *et al.* (2017) asked participants not only how concerned they were in this moment (during the study), but also how relevant the topic (potential health effects from EMF) was in their daily lives. They found differences between those that were “enduringly concerned” (high concern in the questionnaire and high thematic relevance, 13% of the total sample) and those that were “not enduringly concerned” (high concern in the questionnaire, but no high thematic relevance, 30% of the total sample). Those participants who were enduringly concerned considered EMF-exposure to a higher extent as a moral and affective issue, saw themselves as more exposed, and had a less elaborated concept on how EMF-exposure impacts health (e.g., they were more likely to believe that even low exposure can have detrimental effects on health and less likely to agree with the principle “the dose makes the poison”). These findings can be related to a concept by Zwick (2005) who states that there is a difference between “pervasive risks” (those that are permanently perceived as risks) and “switching risks” (those that are only perceived as risks after an external trigger and are otherwise not relevant in the everyday life). Risks can consequently be “switched on” by, for example, news reports (e.g., about new technologies like 5G), conversations with friends or family or on social media, or by questions in a study context.

This is important because recent studies on RP ( Wiedemann *et al.*, 2017) and exposure perception ( Link *et al.*, 2023) regarding RF-EMF from MC-technology have shown that for many people, the topic is not very relevant in their everyday lives. According to Zwick (2005) this would not necessarily mean that study participants could not express concerns or report high RP.

Transferring these findings to the field of precautionary information, it is noteworthy that none of the previous studies on the effects of precautionary information has considered relevance of the topic as a moderating factor. Studies so far have been conducted with student or general population samples without considering how likely those people are to look for or come across precautionary information in their daily lives. It is plausible to assume that people who regularly think about the topic “EMF and health” or who find information about it generally interesting are more likely to come in touch with precautionary information, for example when they come across, for example in articles or online. This has important practical implications, because communication effects can only become practically relevant in people that actually come across the information in real life. It is possible that in a study context, the effects of precautionary information on RP and trust are stronger for those people with lower relevance of the topic, because even though they likely had a lower RP and little engagement in precaution initially but might feel momentarily threatened when they learn about the scientific uncertainties and the existence of precautionary measures. In other words, this study attempts to test whether the effects reported in former studies (e.g., Boehmert *et al.*, 2020) are of any practical relevance.

### Research questions and hypotheses

In this study, we research the effects of precautionary information on RP and trust in a large general population sample, using information that focused on individual mobile phone use. Furthermore, we investigate whether personal relevance of the topic moderates the effect of precautionary information.

As most previous studies found that precautionary information led to higher RP than basic information, we expected to find this effect as well, however, only if participants assume that they take no precautionary measures.

*Hypothesis 1: There is a significant influence of information type on RP. After reading a text with precautionary information (compared to a text with basic information only)…*

*a) affective RP regarding RF-EMF from mobile communications and b) general RP regarding RF-EMF emitted by mobile phones if participants assume that no precautionary measures are taken (CR1) are higher, but c) general RP regarding RF-EMF emitted by mobile phones is not higher if participants assume that precautionary measures are regularly taken (CR2).*

Effects of precautionary recommendations on trust have been found in some studies ( Wiedemann & Schütz, 2005), but not in others ( Wiedemann *et al.*, 2006) or only in parts of the sample ( Boehmert *et al.*, 2017). We expected to find an effect as large samples allow us to discover small effects.

*Hypothesis 2: There is a significant influence of information type on trust in national institutions of radiation protection. Trust is lower after reading the text with precautionary information (compared to the basic text).*

Regarding personal relevance, we investigated if it moderates the effect of precautionary information on RP and trust. The findings by Boehmert and colleagues ( Boehmert, 2018; Boehmert *et al.*, 2016; Boehmert *et al.*, 2017) indicate that precautionary information has a stronger effect on people with low trait-anxiety. We expected the influence of precautionary information to depend on perceived personal relevance of the topic, with a lower influence if relevance is higher.

*Hypothesis 3: The influence of information type on the dependent variables (increase of RP and decrease of trust) is moderated by the personal relevance of the topic “mobile phone radiation and health”. At higher relevance, the influence of precautionary information on a) affective RP, b) CR1, and c) trust is lower than at lower relevance.*

We didn’t expect an effect of precaution on CR2, consequently it is not included in *H*3.

As the study was conducted in Germany and Greece, it is possible to explore country comparisons. These countries were chosen because when researching RP regarding RF-EMF, it is interesting to look at two countries where RP is initially quite different. According to the Eurobarometer 2010 ( TNS Opinion & Social, 2010) the percentage of citizens concerned about EMF is much higher in Southern Europe (e.g. Greece: 81%) than in middle and Northern Europe (e.g. Germany: 24%). The high percentage of Greek citizens concerned about EMF was also confirmed in 2018 according to the results of a nationwide public opinion survey about perceptions, attitudes and knowledge on radiation-related topics ( EEAE, 2018).

## Methods

### Sample

To determine the sample size necessary for this study, a power analysis with G-Power was performed. It was assumed that linear multiple regression (LMR) analyses with up to six predictors would be used, with a significance level of .05, a power of .8 and the aim to detect small effects. This resulted in a sample size of *N* = 688 participants, which was rounded up to a target sample size of *N* = 700 in each country. In the process of recruiting the final participants, this target was slightly overachieved, see below.

We aimed to recruit a heterogenous sample, using interlocking quotas for age and gender. To create the quotas while maintaining anonymity, six age ranges were defined (18–29, 30–39, 40–49, 50–59, 60+). The proportions of the population to create the quota were based on data from Eurostat. In the final stage of data collection, it turned out that particularly in Greece, quotas for older people (especially females) were impossible to fill, consequently we increased participant numbers for the bordering quotas. For interpretation, it therefore needs to be considered that the older population is underrepresented. The second quota was the "personal relevance of the topic", which was measured by showing participants the teaser of an article on the topic of MC and health and asking them how likely they would decide to read this article, assuming they have time (7-point Likert scale). The quota was used to ensure that the distribution of relevance is roughly balanced (about 1/7 on each point of the scale) to have a diverse sample regarding relevance. In the final sample, each of the seven scale points was represented with 91 to 110 participants. In Germany, it was easier to fill the quotas representing lower relevance (1-3), in Greece the other way around (5-7), which was an indication that relevance of the topic is higher in Greece compared to Germany.

A market research company was assigned to recruit the samples, participants were panel members who fulfilled the general entry criteria (being at least 18 years old and having not participated in previous studies about RF-EMF by the same researchers). They were contacted by the panel provider by email, received a link to the study, and were incentivized by collecting points/money from the panel provider.

While over 5000 potential participants clicked on the link, many of them did not start the study or were screened out for different reasons. Planned reasons for exclusions after starting the study were full quotas, failed attention checks, speeding or long inactivity. Due to missing data because of a small programming mistake, which was noticed after the soft launch, 9 participants had to be excluded at a later point. Table 1 shows the numbers of participants excluded per reason. The final sample after exclusions consisted of *N* = 727 participants in Germany (345 basic group, 382 precaution group) and *N* = 721 (350 basic group, 371 precaution group) participants in Greece.

**Table 1.** Sample and exclusions.

	Germany	Greece
Clicked on the study	2071	3021
Did not start participation	35	47
Full quota	981	1588
Inactivity for 30+ minutes or interrupted responding	65	62
“Speeding” (participation time of less than 3 minutes)	71	39
Failed attention check	76 and 108	314 and 244
**Sum of “planned” exclusions**	1335 (735 left)	2294 (727 left)
Excluded because of missing data (only during soft launch)	5	4
Excluded from analysis because “diverse” or “other” in gender	3	2
**Final sample for analysis**	727	721

### Study preparation and data collection

The translation of the study material from German to Greek was done by professional translators and checked by native speaking EMF experts. Pre-Tests were conducted to test initial reactions, identify unclear formulations or misunderstandings and increase usability. In Germany, *n*=14 and in Greece, *n*=4 qualitative pre-tests were performed, where participants spoke their thoughts out loud while completing the study. Later, quantitative pre-tests were performed in Germany. The study was designed to take a maximum of ten minutes to complete, average completion time was *M* = 7.6 minutes ( *SD* = 4.1) in Germany and *M* = 7.2 ( *SD* = 3.7) minutes in Greece. Data were collected in March/April 2024 in form of an online questionnaire on the platform Unipark.

### Conditions

The design of the study was between-subject with one factor, text type. Participants received either 1) a basic text with general information about RF-EMF and health, or 2) the same basic text with additional information on precautionary measures for reducing personal exposure to RF-EMF regarding usage of a mobile phone. They were randomly assigned to the different conditions; the random allocation was done automatically by the platform Unipark. In addition, the precautionary information group was once more split into two groups, where the information was either formulated as an information (2a), or as recommendation (2b). Comparing these two versions is not part of the present paper but of a separate publication, however it should be noted that there were no significant differences between groups 2a and 2b. The final number of participants per condition in both countries is shown in Table 2.

**Table 2.** Number of participants per country and condition.

	Number of participants	Percent		Number of participants	Percent
Germany			Greece		
	727	100%		721	100%
Basic text	345	47,5%		350	48,5%
Precaution text	382	52,5%		371	51,5%
-Recommendation	184	25,3%		181	25,1%
-Information	198	27,2%		190	26,4%

### Procedure of the study

**Figure 1.**
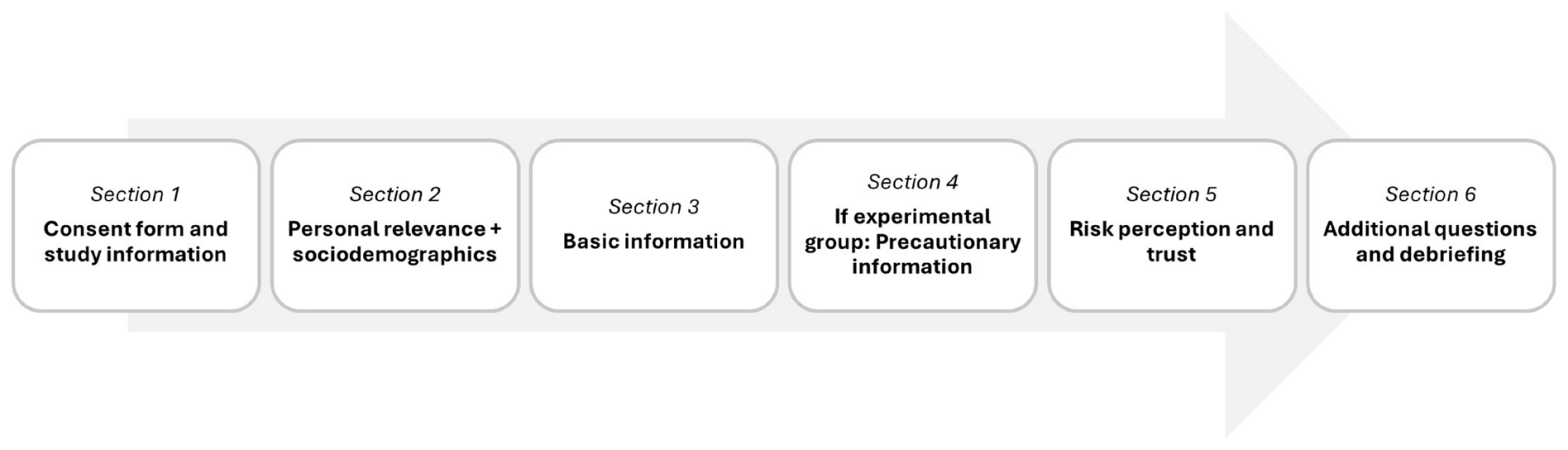
Procedure of the study.

The different steps of the study are shown in Figure 1. After reading the study information and giving consent to take part in the study, personal relevance of the topic “RF-EMF and health” was captured. Then, participants received a basic information text and, only if they were in the corresponding condition, a precautionary information text. There was a timer on each text page that allowed continuation only after 30 seconds. Afterwards, participants responded to several questions, particularly regarding their RP and trust. Finally, they were debriefed and thanked for participation.

Participants received two attention check questions, not passing led to immediate exclusion from the study. The first attention check was placed after the information texts and asked: “Which term was abbreviated with ‘EMF’ in the text?”, which had been explained previously. There were four response options, which could all in theory be abbreviated to “EMF”, the only correct one being “Electromagnetic Fields”. The second attention check was embedded in the block of questions on trust, where participants were asked to “Please check the box ‘strongly agree’”.

### Information texts

All information in this study was based on what the German Federal Office for Radiation Protection (BfS) and the Greek Atomic Energy Commission (EEAE) present on their websites, adapted by us to make the information appropriate for a short experiment. In the basic text, participants were first told that the information is provided by the “German/Greek radiation protection agency”, which is a fictional agency. Naming a fictional institution as information source was chosen to avoid any influence of prior attitudes or knowledge about the existing institutions.

In the basic text, it was first explained what EMF are and how MC works, mentioning also different MC-standards like 5G. Regarding possible health effects the text informed that there is no evidence for negative effects below the statutory limits, but that scientific uncertainties remain, particularly for the recent technological developments (5G). In the precaution text, information on possible measures to reduce personal exposure to RF-EMF when using mobile phones was given. A total of six precautionary measures was introduced, e.g., keeping phone calls short and making no calls when the reception is poor. The exact wording differed between the information version (using terms like “inform” and “precautionary measures”) and the recommendation version (using terms as “advice” and “precautionary tips”). The experimental material can be found in the connected OSF-project ( https://doi.org/10.17605/OSF.IO/Z7UV3).

### Measures and dependent variables

**Figure 2.**
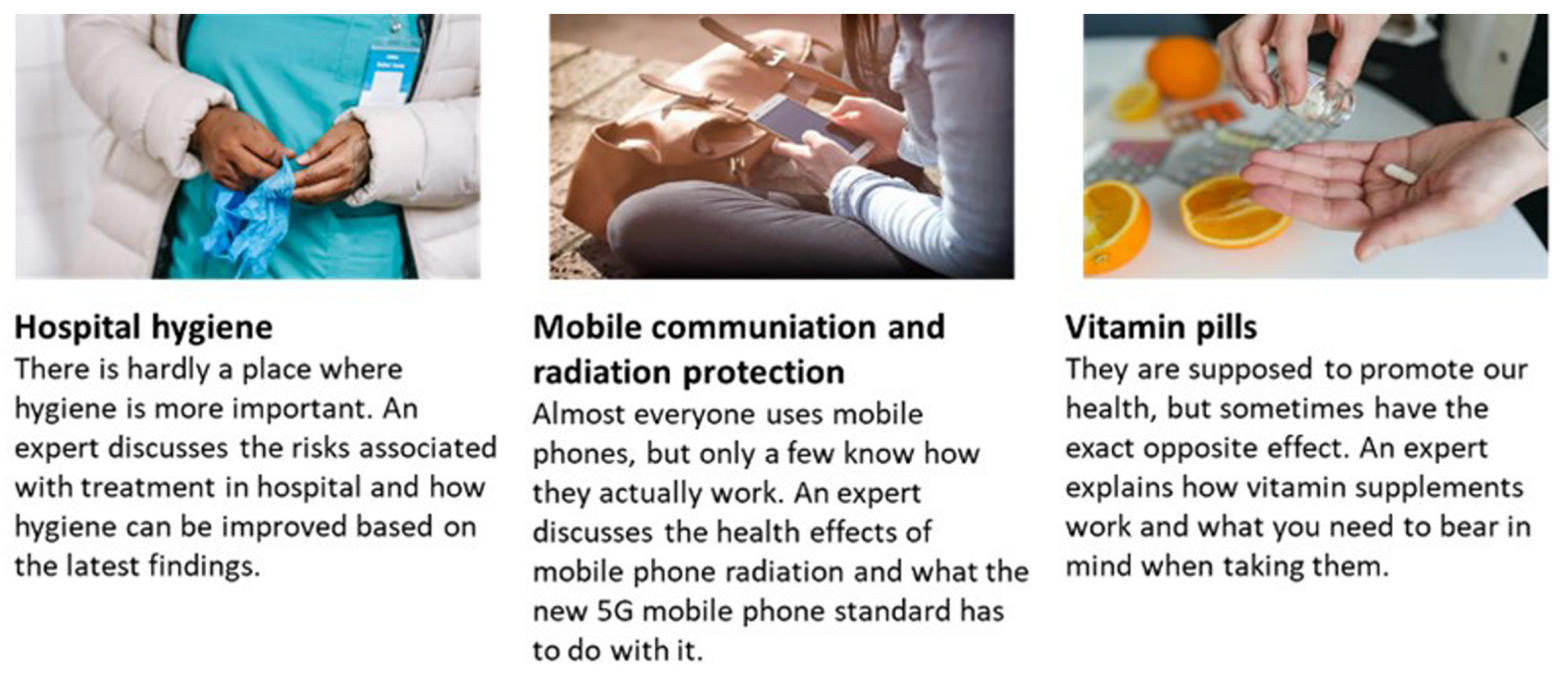
Articles for the practical relevance question.

***Personal relevance of the topic.*** Personal relevance of the topic “mobile phone radiation and health” was assessed in two different ways. As a novel approach to capture *practical relevance,* which was supposed to capture a general interest, resulting in a higher likelihood that someone will come across information on the topic in their daily lives, participants were shown three article suggestions with a title and two descriptive sentences (see Figure 2) that all resolved around health-related topics, one of them being titled “mobile communication and radiation protection”. They responded on a scale from 1 (very unlikely) to 7 (very likely) how likely they would click on each of the articles to read them, assuming that they have to wait a while and consequently have some time. The other two articles, which were about “hospital hygiene” and “vitamin pills”, were just included to make the scenario more realistic and add a reference if necessary. This was followed by one explicit question on *thematic relevance* (“How often in your daily life do you think about the topic ‘mobile phone radiation and health’”? – from 1 = never to 7 = very often) which had already been used by Wiedemann *et al.* (2017).

***Risk perception.*** *Affective RP* was measured with six items, based on Walpole and Wilson (2021). Participants were asked how *concerned*, *worried* and *afraid* they are “ *because of the EMF emitted by your mobile phone/mobile phone base stations*” (1 = not at all, 7 = very much). ( *General) conditional RP* was measured under two conditions (based on Boehmert *et al.*, 2016): if taking no precautionary measures (CR1) (described as “ *measures you can take to do the following: a) reduce the duration of your mobile phone use (e.g., keep phone calls short) and b) increase the distance from the mobile phone (e.g., use a headset when making calls)*” and if taking such measures (CR2). Participants were asked how dangerous they think the EMF from mobile phones are, responding on a scale from 1 (not dangerous at all) to 7 (very dangerous).

***Trust.*** *Trust in national institutions of radiation protection* was queried by using five adapted items from the scale “trust in the scientific community” ( Nisbet *et al.*, 2015). Participants were shown statements and asked how strongly they agree with them, for example: *“Information from national institutions of radiation protection, e.g., the German/Greek radiation protection agency, is trustworthy”*.

***Additional variables.*** Besides the measures relevant for hypothesis testing, more items were included for exploratory research. *Self-efficacy* regarding precaution and *perception of consistency* of the text were assessed at the end of the study with one item each by asking how strongly participants agree with a statement *. Further variables that were measured but are not included in the analyses of the current study are exposure perception*, *susceptibility* (perceived likelihood of negative consequences) and *severity* (of these consequences) were measured with items adapted from Walpole and Wilson (2021) for both mobile phones and base stations after the questions on affective RP.

### Analysis methods

To test for differences between the basic text and the precaution group ( *H*1+ *H*2), we performed LMR analyses with text group and gender (dummy-coded, with 1 referring to both the precautionary information group and males) as predictors, without considering other possible influencing factors. Outcomes were the measures of affective and conditional RP, as well as trust and analyses were conducted separately for each DV. This resulted in four computed regression models for each country. The regression equation had the form:

DV = *b*_0_ + *b*_1_ x Text + *b*_2_ x Gender.

To include the influence of personal relevance ( *H*3), LMR analyses were performed with text group and gender (again dummy-coded), practical and thematic relevance (z-standardized, as recommended by Aiken *et al.* (1991) and the interactions between text group and each of the relevance measures. Outcomes were again affective and conditional RP (only CR1), as well as trust, analyses were conducted separately for each DV. This resulted in three computed regression models for each country. The regression equation had the form:

DV = *b*_0_ + *b*_1_ x Text + *b*_2_ x Gender + *b*_3_ x Practical Relevance + *b*_4_ x Text x Practical Relevance x *b*_5_ x Thematic Relevance + *b*_6_ x Text x Thematic Relevance.

The study was pre-registered prior to data collection under https://osf.io/8cqrf/. Minor deviations from the preregistration are described in detail in Table 3.

**Table 3.** Preregistration deviations.

Details	Original Wording	Deviation Description	Reader Impact
Type: Adjustment of a part of *H*1 Reason: Due to the high correlation we decided to summarize the items on affective RP regarding mobile phones and base stations instead of treating them separately. Also, wording was slightly changed to shorten the hypotheses. Timing: After data access	(…) (a) affective RP regarding RF-EMF emitted by mobile phones is higher. (b) affective RP regarding RF-EMF emitted by mobile phone base stations is higher. (c) general RP regarding RF-EMF emitted by mobile phones is higher if participants assume that no precautionary measures are taken (CR1). (d) general RP regarding RF-EMF emitted by mobile phones is not higher if participants assume that precautionary measures are regularly taken (CR2).	(…) a) affective RP regarding RF-EMF from mobile communications and b) general RP regarding RF-EMF emitted by mobile phones if participants assume that no precautionary measures are taken (CR1) are higher, but c) general RP regarding RF-EMF emitted by mobile phones is not higher if participants assume that precautionary measures are regularly taken (CR2).	Summarizing the items on affective RP should make the analysis easier to follow because we have one DV less. The changes in wording have no impact on the analysis and should make the paper more understandable for readers.
Type: Change in wording in a part of *H*3. Reason: Improve understanding for readers, specification about DVs. Timing: After data access.	(…) At higher relevance, the influence of precautionary information on risk perception and trust is lower than at lower relevance.	(…) At higher relevance, the influence of precautionary information on a) affective RP, b) CR1, and c) trust is lower than at lower relevance.	Better understanding of the results as it is now clearly distinguished which DV we are referring to.

## Results

### Data preparation and descriptive analyses

Data analysis was performed with the statistics program JASP (version 0.19.1.0) and with Libre Office. Items with different polarity were recoded and scales were combined into indices. As affective RP regarding mobile phones and base stations turned out to correlate high ( *r* = .82), we combined them into a single factor, using principal component analysis.

Internal consistency was good for trust in national institutions of radiation protection (five items, Cronbachs alpha >.8), consequently a scale mean was calculated. The correlation between practical and thematic relevance was calculated and as it was only *r* = .6, the two measures were considered as two different predictors.

Differences regarding gender were analysed with ANOVAs to determine if it should be included in the LMR models as predictor. In Germany, there were significant gender differences for affective RP and for CR1 with females indicating higher RP than males. In Greece, there were significant gender differences for all RP measures, with females indicating higher RP than males (for details see Table 4). Gender was therefore included in the LMR analyses.

**Table 4.** Descriptive data on dependent variables separated by gender.

Germany (n = 363 male, n = 364 female)								
	Affective RP		CR1		CR2		Trust	
	*M*	*F*	*M*	*F*	*M*	*F*	*M*	*F*
Mean	2.58	2.81	3.25	3.53	2.59	2.7	4.75	4.56
SD	1.37	1.5	1.44	1.6	1.29	1.4	1.43	1.32
Difference	*F*(1,725)=4.77 *p*=.03*		*F*(1,725)=6.14 *p*=.01*		*F*(1,725)=1.18 *p*=.28		*F*(1,725)=3.79 *p*=.05	
Greece (n = 419 male, n = 302 female)								
Mean	3.63	4.3	4.3	4.83	3.13	3.69	4.17	4.1
SD	1.62	1.53	1.64	1.43	1.56	1.5	1.43	1.2
Difference	*F*(1,719)=31.25 *p*<.001*		*F*(1,719)=20.26 *p*<.001*		*F*(1,719)=23.39 *p*<.001*		*F*(1,719)=0.42 *p*=.52	

Note: Scales on affective RP ranged from 1 (not at all) to 7 (very much), on conditional RP from 1 (not dangerous at all) to 7 (very dangerous), on trust from 1 (do not agree at all) to 7 (totally agree).

### Intercorrelations among variables

The RP-measures had medium to high correlations with each other. Also, practical and thematic relevance correlated positively with the RP-measures, particularly the correlations with thematic relevance were high. Trust correlated negatively with RP and thematic relevance (the latter only in Germany). Table 5 shows all correlations.

**Table 5.** Correlations among relevance measures and dependent variables.

	Practical Relevance	Thematic Relevance	Affective RP	CR1	CR2	Trust
Practical relevance		*r* = 0.57 *p* < .001*	*r* = 0.43 *p* < .001*	*r* = 0.37 *p* < .001*	*r* = 0.28 *p* < .001*	*r* = 0.05 *p* = 0.21
Thematic Relevance	*r* = 0.63 *p* < .001*		*r* = 0.67 *p* < .001*	*r* = 0.53 *p* < .001*	*r* = 0.47 *p* < .001*	*r* = -0.04 *p* = .34
Affective RP	*r* = 0.4 *p* < .001*	*r* = 0.61 *p* < .001*		*r* = 0.76 *p* < .001*	*r* = 0.66 *p* < .001*	*r* = -0.14 *p* = .001*
CR1	*r* = 0.4 *p* < .001*	*r* = 0.52 *p* < .001*	*r* = 0.75 *p* < .001*		*r* = 0.63 *p* < .001*	*r* = -0.11 *p* = .004*
CR2	*r* = 0.28 *p* < .001*	*r* = 0.45 *p* < .001*	*r* = 0.67 *p* < .001*	*r* = 0.71 *p* < .001*		*r* = -0.26 *p* < .001*
Trust	*r* = -0.05 *p* = .15	*r* = -0.19 *p* < .001*	*r* = -0.33 *p* < .001*	*r* = -0.28 *p* < .001*	*r* = -0.33 *p* < .001*	

Note: Upper right triangle: Greece, lower left triangle: Germany.

### Assumption checks for LMR

Prior to the analyses, assumptions for LMR were checked. There were no violations except for the normality assumption in some of the models for *H*1. However, as the normality assumption is robust for large samples ( Eid *et al.*, 2010; Knief & Forstmeier, 2021), we decided to proceed as planned with the LMR analyses. Details can be found in the connected OSF-project ( https://doi.org/10.17605/OSF.IO/Z7UV3).

### Hypotheses 1 and 2: text group (and gender)

Results of the LMR analyses for *H*1 and *H*2 can be seen in Table 6. In Germany, the models for affective RP, CR1 and trust were overall significant, in Greece all models were overall significant. In Germany, text condition was a significant predictor only for CR1 ( *t* = 3.11, *p* = .002). Participants from the precaution group indicated on average a higher RP ( *M* = 3.56, *SD* = 1.51) than participant from the basic text group ( *M* = 3.21, *SD* = 1.53). Gender was a significant predictor for affective RP ( *t* = -2.17, *p* = .03), with females indicating higher RP ( *M* = 2.81, *SD* = 1.5) than males ( *M* = 2.58, *SD* = 1.37) and for CR1 ( *t* = -2.46, *p* = .01), again with females indicating higher RP ( *M* = 3.53, *SD* = 1.6) than males ( *M* = 3.25, *SD* = 1.44). All other regression weights were not significant.

**Table 6.** Results of the LMR-analyses for H1+H2.

Germany (n = 345 basic text, n = 382 precaution text)								
	Affective RP		CR1		CR2		Trust	
	*B* ( *SE*)	*p*	*B* ( *SE*)	*p*	*B* ( *SE*)	*p*	*B* ( *SE*)	*p*
Intercept	0.02		3.35		2.67		4.47	
Text	0.12 (0.07)	.12	0.35 (0.11)	.002*	0.06 (0.1)	.58	0.16 (0.1)	.12
Gender	-0.16 (0.07)	.03*	-0.28 (0.11)	.014*	-0.12 (0.1)	.28	0.2 (0.1)	.05
Multiple *R* ^2^	0.01		0.02		0.002		0.01	
*F* ( *p*)	3.61 (.03)		7.93 (<.001)		0.74 (.48)		3.11 (.045)	
Greece (n = 350 basic text, n = 371 precaution text)								
Intercept	0.21		4.71		3.61		4.0	
Text	0.05 (0.07)	.52	0.22 (0.12)	.06	0.14 (0.11)	.24	0.19 (0.1)	.06
Gender	-0.41 (0.07)	<.001*	-0.52 (0.12)	<.001*	-0.55 (0.12)	<.001*	0.08 (0.1)	.45
Multiple *R* ^2^	0.04		0.03		0.03		0.01	
*F* ( *p*)	15.63 (<.001)		11.91 (<.001)		12.40 (<.001)		2.03 (.13)	

Note: Text group and Gender were dummy coded with 1 referring to both the precautionary information group and males.

In Greece, text condition was not a significant predictor for any of the outcomes. Gender was a significant predictor for all RP measures, with females indicating higher RP than males (affective RP: *t* = -5.51, CR1: *t* = -4.34, CR2: *t* = -4.76, all *p* < .001, for means and standard deviations see Table 7). All other regression weights were not significant.

**Table 7.** Descriptive data on dependent variables separated by conditions.

Germany (n = 345 basic text, n = 382 precaution text)								
	Affective RP		CR1		CR2		Trust	
	Basic	Prec.	Basic	Prec.	Basic	Prec.	Basic	Prec.
*M*	2.61	2.77	3.21	3.56	2.61	2.67	4.57	4.73
*SD*	1.38	1.48	1.53	1.51	1.3	1.38	1.43	1.32
Greece (n = 350 basic text, n = 371 precaution text)								
*M*	3.85	3.97	4.4	4.64	3.27	3.44	4.04	4.23
*SD*	1.61	1.63	1.62	1.52	1.56	1.55	1.4	1.24

Note: Scales on affective RP ranged from 1 (not at all) to 7 (very much), on conditional RP from 1 (not dangerous at all) to 7 (very dangerous), on trust from (not agree at all) to 7 (totally agree).

### Hypothesis 3: text group, personal relevance (and gender)

**Figure 3.**
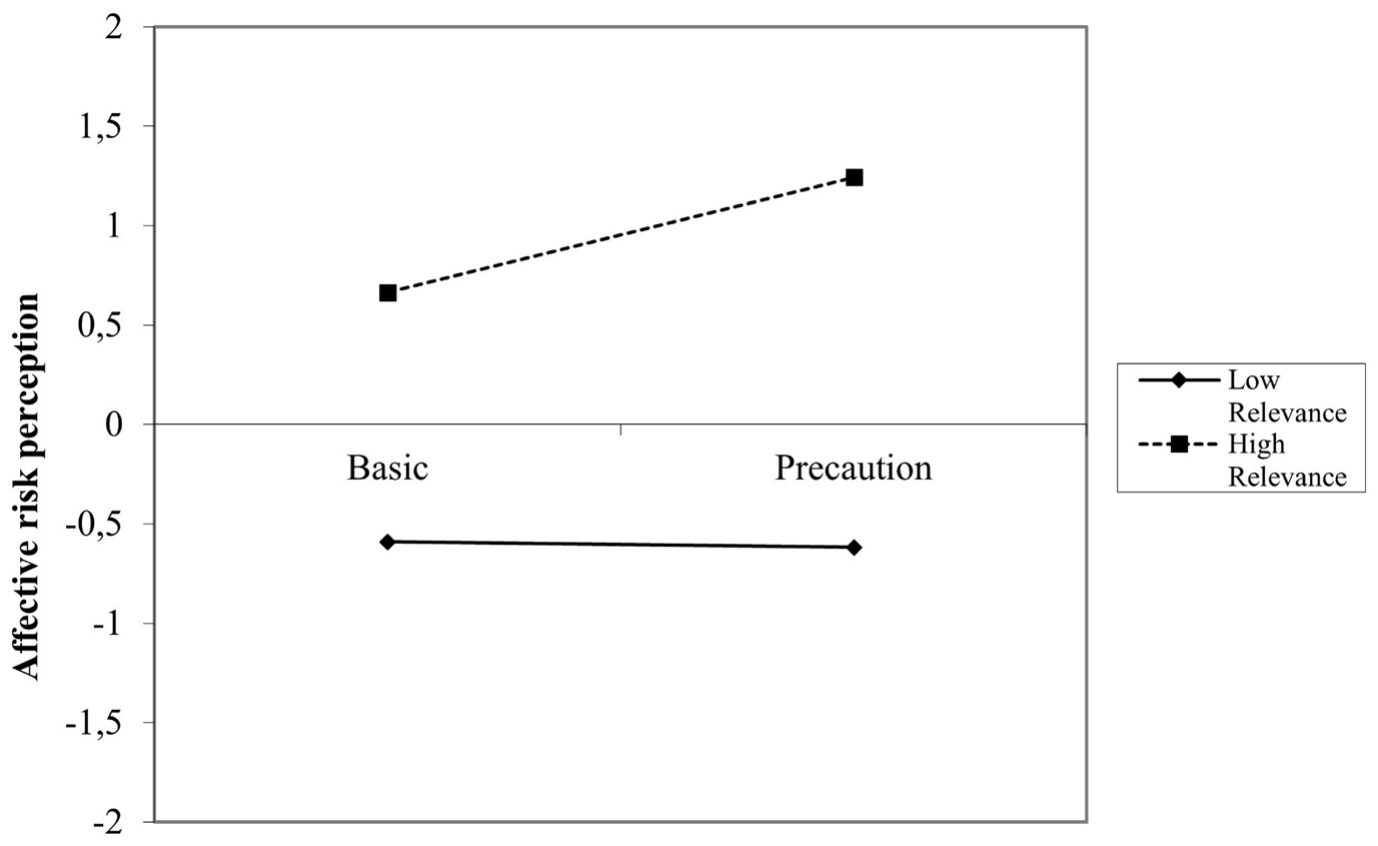
Simple slopes for the interaction between text condition and thematic relevance on affective RP (Germany).

Results of the LMR analyses for *H*3 can be seen in Table 8. In both Germany and Greece, all regression models were overall significant. In Germany, affective RP was significantly predicted by text group (higher RP in the precaution group, *t* = 2.73, *p* = .007), thematic relevance (higher RP going along with higher relevance, *t* = 9.34, *p* < .001), and the interaction thematic relevance*text group ( *t* = 2.13, *p* = .03). Gender, practical relevance and the interaction practical relevance*text group were insignificant predictors in this model. When looking at the simple slopes ( Figure 3) it was shown that at high thematic relevance, the effect of precautionary information compared to basic information was higher than at low thematic relevance (contrary to the expectations). The simple slope test for high relevance was significant ( *t* = 3.48, *p* = .001), while the simple slope for low relevance was insignificant ( *t* = -0.15, *p* = .89).

**Table 8.** Results of the LMR-analyses for H3.

	Affective RP		CR1		Trust	
	*B ^1^*	*p*	*B*	*p*	*B*	*p*
Germany (n = 345 basic text, n = 382 precaution text)						
Intercept	-0.03		3.39		4.5	
Text	0.16 (0.06)	.007*	0.41 (0.1)	<.001*	0.14 (0.1)	.16
Gender	-0.01 (0.06)	.09	-0.22 (0.1)	.02*	0.16 (0.1)	.11
Practical Rel.	0.1	.07	0.15	.01*	0.12	.08
Text*Pract. Rel.	-0.12 (0.08)	.07	-0.1 (0.12)	.41	-0.04 (0.13)	.73
Thematic Rel.	0.5	<.001*	0.41	<.001*	-0.28	<.001*
Text*Them. Rel.	0.16 (0.08)	.03*	0.13 (0.12)	.3	0.1 (0.13)	.48
Multiple *R* ^2^	0.38		0.3		0.05	
*F* ( *p*)	74.28 (<.001)		52.37 (<.001)		6.04 (<.001)	
Greece (n = 350 basic text, n = 371 precaution text)						
Intercept	0.14		4.62		3.99	
Text	0.02 (0.06)	.7	0.19 (0.1)	.06	0.2 (0.09)	.047*
Gender	-0.27 (0.06)	<.001*	-0.34 (0.1)	<.001*	0.07 (0.1)	.48
Practical Rel.	0.08	.11	0.03	.61	0.17	.01*
Text*Pract. Rel.	-0.02 (0.07)	.83	0.2 (0.12)	.1	-0.15 (0.12)	.2
Thematic Rel.	0.57	<.001*	0.47	<.001*	-0.18	.01*
Text*Them. Rel.	0.09 (0.07)	.19	-0.04 (0.12)	.75	0.21 (0.12)	.08
Multiple *R* ^2^	0.47		0.3		0.02	
*F* ( *p*)	104.8 (<.001)		51.69 (<.001)		2.2 (.04)	

Note: Text group and Gender were dummy coded with 1 referring to both the precautionary information group and males. ^1^ Standardized beta for continuous predictor (relevance), unstandardized B (with SE) for dummy-coded predictors.

CR1 was significantly predicted by text group (higher RP in the precaution group, *t* = 4.3, *p* < .001), gender (higher RP in females, *t* = -2.25, *p* = .02) practical relevance (higher RP going along with higher relevance, *t* = 2.59, *p* = .01) and thematic relevance ( *t* = 7.16, *p* < .001). The interactions weren’t significant predictors in this model.

In the model for trust, the only significant predictor was thematic relevance (higher relevance going along with lower trust, *t* = -4.18, *p* < .001).

In Greece, for the models on affective RP and CR1, gender (affective RP: *t* = -4.76; CR1: *t* = -3.34; both *p* < .001) and thematic relevance (affective RP: *t* = 11.5; CR1: *t* = 8.41; all p < .001) were the only significant predictors, RP being higher in females and higher RP going along with higher thematic relevance. For trust, text condition ( *t* = 2.0, *p* = .047) was a significant predictor, trust being higher in the precaution group. Also, practical relevance ( *t* = 2.48, *p* = .01), and thematic relevance ( *t* = -2.67, *p* = .01) were significant predictors, higher practical relevance going along with higher trust and higher thematic relevance going along with lower trust. There were no interaction effects.

### Exploratory analyses

Self-efficacy was significantly higher in the precaution group than in the basic group in both Germany (basic: *M* = 4.54, *SD* = 1.69; precaution: *M* = 5.25, *SD* = 1.4; *t* = -6.14, *p* < .001) and Greece (basic: *M* = 4.61, *SD* = 1.62; precaution: *M* = 4.88, *SD* = 1.59; *t* = -2.33, *p* = .02). Perceived consistency of the information was also higher in the precaution group than in the basic group, in both Germany (basic: *M* = 5.06, *SD* = 1.39; precaution: *M* = 5.42, *SD* = 1.35; *t* = -3.49, *p* < .001) and Greece (basic: *M* = 5.07, *SD* = 1.35; precaution: Greece: *M* = 5.31, *SD* = 1.35, *t* = -2.41, *p* = .02). Regarding country differences, relevance and RP were significantly higher in Greece compared to Germany.

## Discussion

### Interpretation of the results and methods

In this study, the influence of precautionary information and personal relevance of the topic on RP and trust was investigated. The risk topic was RF-EMF emitted by mobile communication technology.

To look at the influence of the different texts (basic vs. precautionary information) on RP and trust, regression models with only condition and gender as predictors were conducted. Contrary to *H*1a (influence of text group on affective RP), there was no significant effect of text group on affective RP. In line with *H*1b and *H*1c, conditional RP in Germany was significantly higher in the precaution group compared to the basic group, but only if assuming that no precautionary measures are taken. As expected, if assuming that precautionary measures are taken, there was no difference. Contrary to the hypotheses, in Greece, there were no text group differences at all. Contrary to *H*2, the precautionary information did not have an effect on trust, neither in Germany nor in Greece. 

In the next step, regression models with condition, gender, practical and thematic relevance, as well as the interactions between condition and each relevance variable were conducted. The predicted interaction ( *H*3) was not found in any of the models. In Germany, the interaction between thematic relevance and condition was significant for affective RP. However, this effect was countervailing as the simple slopes showed a larger effect of precautionary information on affective RP if thematic relevance was higher. Thematic relevance was a significant predictor in all models in both countries, higher relevance going along with higher RP and lower trust. In Germany, affective RP was significantly higher in the precaution group in the models that included relevance. In Greece, trust was significantly higher in the precaution group in the models that included relevance. A summary of the hypothesis tests and their outcomes can be found in Table 9.

**Table 9.** Summary of decisions about hypotheses.

		Specific Hypothesis ^1^	Decision Germany	Decision Greece
*H*1	a	Affective _precaution_ > Affective _basic message_	Keep *H*_0_^2^	Keep *H*_0_
	b	CR1 _precaution_ > CR1 _basic message_	**Reject *H*_0_**	Keep *H*_0_
	c	CR2 _precaution_ = CR2 _precaution_	**Reject *H*_0_**	**Reject *H*_0_**
*H*2		Trust _precaution_ < Trust _basic message_	Keep *H*_0_	Keep *H*_0_^2^
*H*3	a	Relevance is moderator for affective RP	Keep *H*_0_	Keep *H*_0_
	b	Relevance is moderator for CR1	Keep *H*_0_	Keep *H*_0_

Note: ^1^CR1 = conditional risk perception without precautions, CR2 = conditional risk perception with precautions. ^2^ Was not significant in the analysis with text group and gender as predictors, but significant in the analysis with the relevance measures.

Our results differ from those of previous studies which found that RP was higher and trust lower when receiving precautionary information compared to basic information only (e.g., Boehmert *et al.*, 2020; Wiedemann & Schütz, 2005; Wiedemann *et al.*, 2006). There are different possible explanations for these findings. First it needs to be noted that the effect of precautionary information was not found in all previous studies. For example, Boehmert, Wiedemann, and Croft (2016) did not find a general difference, only if considering individual variables, like trait-anxiety and gender. Most importantly, when comparing research on the effects of precautionary information, it needs to be kept in mind that researchers used different types of precautionary information for their studies. For example, earlier studies (e.g., Wiedemann & Schütz, 2005; Wiedemann *et al.*, 2006) presented mostly precautionary information that are impossible or hard to implement by citizens individually. In our study, as well as in the more recent precursor studies, precautionary information referred to measures that citizens can implement when using their own mobile devices. Consequently, even though precautionary information may lead to people becoming aware that there are uncertainties, and their RP increases as a result, they may feel less helpless and more confident in their ability to control their own exposure to RF-EMF. Our exploratory finding of higher self-efficacy in the precaution group as well as the finding that trust in Greece was higher in the precaution group (though only if including relevance in the analysis) support this possible explanation. In the future, it would be interesting to systematically compare these different kinds of precautionary information regarding their effects on RP and trust.

Of course, there were more differences between our study and previous ones that may have influenced results, like different wording in the information texts and different choices of dependent variables. For example, Wiedemann and colleagues ( Wiedemann *et al.*, 2006; Wiedemann & Schütz, 2005) used the term “electrosmog” while we used “electromagnetic fields” or “mobile communication radiation”. These terms may well lead to different associations and affects in participants. We think our choice of experimental material can be justified well as we used actual government agency communication material as a base for it.

Finally, another potential explanation for the absence of a more pronounced effect of precautionary information might be that RF-EMF as a potential risk triggers less strong responses nowadays than ten or 15 years before. People might nowadays be less ready to increase their RP in response to information on the topic than before. 

Concerning trust, it is important to keep in mind that effects in previous studies have been much less consistent than those on RP. As explained above, there were large differences regarding content but also regarding wording and lengths of the precautionary information not only between our studies and previous ones, but also among the previous ones. All these factors may influence how trustworthy the information is perceived. Furthermore, how trust is measured, and the object of trust could play a role. In previous studies, the object of trust was “public health protection” (e.g., Wiedemann & Schütz, 2005) or “trust in risk regulation” ( Wiedemann *et al.*, 2006). In our study, we asked more specifically for trust in national institutions of radiation protection, naming the source of the information texts as an example. Also, we used a short questionnaire with five items, instead of one item only. It was interesting to see in the analysis that trust is not explained well by any of our predictors. Possibly, trust is something that develops more long-term and is less influenced by reading short texts than RP, which can change momentarily when triggered by something, e.g., information received in the context of a study ( Zwick, 2005).

Unexpectedly, we did not find personal relevance of the topic to be a moderator on the effect of precautionary information on RP or, in the one analysis where we did see an effect, the effect was countervailing. Given that the effect of precautionary information on RP was not very pronounced in the current study, it is generally not surprising that this moderation hypothesis is also not supported. Relevance correlated highly with all RP-measures in both countries and particularly thematic relevance was a significant predictor for explaining individual differences in RP. Consequently, it seems that including relevance as a predictor or covariate when investigating effects on RP is important. For example, the group differences regarding affective RP in Germany and regarding trust in Greece became only visible in our analysis when relevance was included, indicating that only by not considering the variance explained by relevance, the detection of group differences was possible. Practical relevance added additional value in some models (CR1 in Germany, trust in Greece). It is too early to tell if our approach to use general interest in the topic as a proxy for personal relevance is helpful for studying effects of risk communication. While it could capture better what role a topic plays in people’s daily lives, the explicit question on thematic relevance turned out to be the more important predictor in our analyses.

As it had proven to be a good idea in previous studies, we used a conditional measure for general RP. Our results confirm that this differential measure is important when investigating the effects of precautionary information. As expected, results for RP were very different between the conditional measures. We recommend that future researchers use conditional measures as well.

We found some interesting exploratory results in our study. Regarding country differences, both personal relevance and RP were significantly higher in Greece, which is in line with previous findings from, for example, the Eurobarometer. Apparently, the topic of RF-EMF and health is more relevant in people’s daily lives and more critically perceived in Greece than in Germany. Practical relevance of two other health-related topics was higher in Greece as well, indicating that perhaps the interest in health-related topics is generally higher in Greece. However, this should be looked at more systematically in other studies.

We also found some gender differences; however, they were stronger in Greece. In Germany, females reported higher affective RP and general RP under the condition that no precautionary measures are taken. In Greece, females reported higher RP and relevance across all measures.

### Limitations

As our study took place online, attention check items and survey completion time were our only way to control if people paid attention to the questions. As it is usually the case with anonymous studies, there is no guarantee that participants took it seriously or responded honestly. Furthermore, there is no way of knowing why participants responded in a certain way, only qualitative research could give more insight into this.

Because we measured the dependent variables only once, instead of capturing them before and after reading the information, we did not measure individual changes. This decision was made because a pre-information measurement would have drawn participants’ attention to the topic of health risks from MC and could therefore have influenced the uptake of the information. This is a common trade-off in psychological research between pre-post designs and between-subject designs.

### Implications for research and practice

The finding that precautionary information did not result in lower trust compared to basic information may be good news for institutions that inform about MC and health. Even though the group reading precautionary information indicated higher RP in some measures, one could argue that this is not a problem if it doesn’t go along with lower trust. Furthermore, in this study, self-efficacy was higher if receiving the precautionary information, indicating that even though RP may be increased, people felt more that they could control their personal exposure. For communicators, e.g., agencies for radiation protection, it is likely a good idea to focus their precautionary recommendations on such measure that citizens have control over, because this might allow them to make informed decisions without undermining trust. Also, perceived consistency of the text was higher in the precaution group, which is an indication against the assumption that precautionary information leads to higher RP because they are perceived as inconsistent ( Boehmert *et al.*, 2016).

It is also important to keep in mind that information may be perceived differently depending on how relevant the topic is for recipients. Future researchers on this topic may consider including a relevance measure in their studies. Finally, it is important to consider that the perception of risk-related and precautionary information may differ hugely between for example countries, genders, and age groups. Consequently, one cannot necessarily generalize findings from studies with a limited target group to other populations. More studies on comparisons between countries should be considered to deepen our understanding of sociodemographic and intercultural differences in RP and trust.

## Ethics and consent

All procedures in the study were performed in compliance with relevant laws and institutional guidelines and have been approved in form of an ethics approval (01/12/2023) by the ethics commission of the IU International University of Applied Sciences before data collection started (approval number: 121201). The privacy of human subjects was observed and written informed consent was obtained before participants began the experiment. No personal data was collected in the study, basic sociodemographic information was enquired in categories to ensure anonymity throughout. This procedure was approved by the ethics committee previously.

## Declaration of AI assistance

During the preparation of this work the authors used the tools DeepL and ChatGPT in order to translate and improve language. After using these tools, the authors reviewed and edited the content as needed and take full responsibility for the content of the publication.

## Data Availability

Open Science Framework (OSF): Precautionary information and personal relevance. https://doi.org/10.17605/OSF.IO/Z7UV3 ( Eggeling-Böcker *et al.*, 2024). The project contains the following underlying data: “Experimental material.pdf” (the information texts for all conditions used in the study) “Codebook.docx” (the codebook for the data file) “Assumption_checks_LMR.pdf” (detailed information on the assumption checks for the linear multiple regression analyses) “JASP_December24_AnalysisGermany.csv” (data for Germany in standard tabular format) “JASP_December24_AnalysisGreece.csv” (data for Greece in standard tabular format) “JASP_December24_AnalysisGermany.jasp” (data for Germany including statistical analyses in JASP-format, a free statistics program) “JASP_December24_AnalysisGreece.jasp” (data for Greece including statistical analyses in JASP-format, a free statistics program) Data are available under the terms of the Creative Commons Attribution 4.0 International license (CC-BY 4.0). Open Science Framework (OSF): Precautionary information and personal relevance. https://doi.org/10.17605/OSF.IO/Z7UV3 ( Eggeling-Böcker *et al.*, 2024). The project contains the following reporting guidelines: STROBE Checklist ( von Elm *et al.*, 2007) Data are available under the terms of the Creative Commons Attribution 4.0 International license (CC-BY 4.0).
