# Supplementary material for: Does personal relevance moderate communication effects? The example of risk communication about 5G-related electromagnetic fields
Source: Open Res Eur. 2025 May 23;5:13. Originally published 2025 Jan 20. [Version 2] doi: 10.12688/openreseurope.19236.2 (PMC12000800; doi:10.12688/openreseurope.19236.2)
Supplement: Peer review history

# Reviewer report: Reviewer response for version 2

**DOI:** 10.21956/openreseurope.22171.r54507

**Published:** 2025-06-02

**Authors:** Aduwati Sali

**Affiliations:** 1 Universiti Putra Malaysia (UPM), Serdang, Malaysia

The authors have addressed all concerns raised in the previous version. The paper is now in a better form for indexing.

If applicable, is the statistical analysis and its interpretation appropriate?

Yes

Is the study design appropriate and is the work technically sound?

Yes

Is the work clearly and accurately presented and does it engage with the current literature?

Yes

Are the conclusions drawn adequately supported by the results?

Yes

Are sufficient details of methods and analysis provided to allow replication by others?

Yes

Are all the source data and materials underlying the results available?

Yes

Reviewer Expertise:

NA

I confirm that I have read this submission and believe that I have an appropriate level of expertise to confirm that it is of an acceptable scientific standard.

# Reviewer report: Reviewer response for version 1

**DOI:** 10.21956/openreseurope.20820.r50018

**Published:** 2025-04-15

**Authors:** Jordi Mongay Batalla

**Affiliations:** 1 Warsaw University of Technology, Nowowiejska, Warsaw, Poland

In this paper, the authors present a cross-sectional study that investigates the effects of precautionary information on risk perception and trust in the context of 5G, using large general population samples. The authors aim to demonstrate several hypotheses including the trust in national institutions (which provide the precautionary information).

 First of all, I want to point out that I am not a sociologist or an expert on social and/or psychological behaviour. I am an expert on mobile network technology, and I can only offer a review on this matter. The problem is that the paper only superficially touches on the problem of exposure to EMF.

 In the parts of the EMF exposure, I can say that there seems to be a misunderstanding in the authors' approach to the EMF limits. The precautionary advice to reduce exposure to EMF is the provision of the limits of EMF. This means that the precaution is to limit the exposure. In several parts of the text (e.g., in the background of the Abstract), the authors say that there is precautionary advice to do something more than stay within the limits of EMF exposure.

 About the important content of the paper, I have no expertise to understand whether the socilogical study methodology is correct or not, even if it seems quite logical. 

 However, I want to introduce another point to the debate of the misunderstanding of the issue for potential future research. I acquired this opinion after several meetings and discussions with people who believe that EMF is really damaging to health. I think that one of the main problems is the incapacity of the people to distinguish a serious research analysis from a pseudo-research and even, sometimes, fraudulent analysis. In my opinion (but this is not the opinion of an expert in social behaviour), the problem of pseudo-science obfuscates strongly the debate.

If applicable, is the statistical analysis and its interpretation appropriate?

Yes

Is the study design appropriate and is the work technically sound?

Partly

Is the work clearly and accurately presented and does it engage with the current literature?

Yes

Are the conclusions drawn adequately supported by the results?

Partly

Are sufficient details of methods and analysis provided to allow replication by others?

Yes

Are all the source data and materials underlying the results available?

Yes

Reviewer Expertise:

Mobile network technology - Telecommunications

I confirm that I have read this submission and believe that I have an appropriate level of expertise to confirm that it is of an acceptable scientific standard.

# Reviewer report: Reviewer response for version 1

**DOI:** 10.21956/openreseurope.20820.r51566

**Published:** 2025-04-08

**Authors:** Aduwati Sali

**Affiliations:** 1 Universiti Putra Malaysia (UPM), Serdang, Malaysia

The paper presented a study based on a survey to gauge the public's perception on RF-EMF from 5G signals in Germany and Greece.

 Some comments to improve the paper:

 1. What are the frequencies for 5G signals in respective countries? Does the public aware on this matter?

 2. Can the results be correlated with any awareness programs in each country? It will be interesting to see what are the exposures in terms of awareness campaign by relevant bodies in the country which may affect the perception results. 

 3. Is there any work by the authors to support the findings in terms of measurement campaign? Is the radiation exposure in one country higher than the other?

If applicable, is the statistical analysis and its interpretation appropriate?

Yes

Is the study design appropriate and is the work technically sound?

Yes

Is the work clearly and accurately presented and does it engage with the current literature?

Yes

Are the conclusions drawn adequately supported by the results?

Yes

Are sufficient details of methods and analysis provided to allow replication by others?

Yes

Are all the source data and materials underlying the results available?

Yes

Reviewer Expertise:

Mobile and satellite communications, IoT, radiation exposure.

I confirm that I have read this submission and believe that I have an appropriate level of expertise to confirm that it is of an acceptable scientific standard, however I have significant reservations, as outlined above.

## References

[1] : An Assessment of Extrapolated Field Strengths Versus Distance, Measurement Time, and Induced Traffic From 5G Base Station in C-Band. *IEEE Access* .2024;12: 10.1109/ACCESS.2024.3448257 130639-130653 10.1109/ACCESS.2024.3448257

# Reviewer report: Reviewer response for version 1

**DOI:** 10.21956/openreseurope.20820.r51562

**Published:** 2025-03-13

**Authors:** Adedotun Temitope Ajibare

**Affiliations:** 1 University of Cape Town, Rondebosch, Western Cape, South Africa; 2 Electrical Engineering, NewBridge Graduate Institute, Tygervalley, Western Cape, South Africa

The article has academic merit, the topic is very important in knowing the perspective of telecommunication users regarding, risk perception, trust, RF-EMF exposure and their health.

 The authors investigated the risk perception and trust of mobile users using personal relevance and precautionary information.

 The experimental design and methods are adequate, the results are well discussed and presented accurately.

**Concern**: 5G should be defined before the use as an acronym.

 After discussing the result, I believe there should be a section/subsection to conclude the research. i.e., Conclusion section (summarising the research) is missing.

If applicable, is the statistical analysis and its interpretation appropriate?

Yes

Is the study design appropriate and is the work technically sound?

Yes

Is the work clearly and accurately presented and does it engage with the current literature?

Yes

Are the conclusions drawn adequately supported by the results?

Yes

Are sufficient details of methods and analysis provided to allow replication by others?

Yes

Are all the source data and materials underlying the results available?

Partly

Reviewer Expertise:

5G and beyond networks, radio resource management in wireless networks, network optimization, radiofrequency radiation, data analytics, computational intelligence, and machine and reinforcement learning.

I confirm that I have read this submission and believe that I have an appropriate level of expertise to confirm that it is of an acceptable scientific standard.
